# The Relationship between Air Pollution and Depression in China: Is Neighbourhood Social Capital Protective?

**DOI:** 10.3390/ijerph15061160

**Published:** 2018-06-02

**Authors:** Ruoyu Wang, Desheng Xue, Ye Liu, Penghua Liu, Hongsheng Chen

**Affiliations:** 1 School of Geography and Planning, Sun Yat-Sen University, Xingang Xi Road, Guangzhou 510275, China; wangry6@mail2.sysu.edu.cn (R.W.); eesxds@mail.sysu.edu.cn (D.X.); liuph3@mail2.sysu.edu.cn (P.L.); 2 Guangdong Key Laboratory for Urbanization and Geo-simulation, Sun Yat-Sen University, Xingang Xi Road, Guangzhou 510275, China; 3 School of Architecture, Southeast University, Si-Pai-Lou Road No. 2, Nanjing 210096, China; hongsheng.chen2006@163.com

**Keywords:** PM_2.5_ concentrations, depressive symptoms, neighbourhood social capital, moderate effect, China

## Abstract

There is increasing evidence from the developed world that air pollution is significantly related to residents’ depressive symptoms; however, the existence of such a relationship in developing countries such as China is still unclear. Furthermore, although neighbourhood social capital is beneficial for health, whether it is a protective factor in the relationship between health and environment pollution remains unclear. Consequently, we examined the effects of cities’ PM_2.5_ concentrations on residents’ depressive symptoms and the moderating effects of neighbourhood social capital, using data from the 2016 wave of China Labourforce Dynamics Survey and the real-time remote inquiry website of Airborne Fine Particulate Matter and Air Quality Index. Results showed that PM_2.5_ concentrations and neighbourhood social capital may increase and decrease respondents’ depressive symptoms, respectively. Notably, neighbourhood social capital decreased the negative effect of PM_2.5_ concentrations on respondents’ depressive symptoms. These analyses contributed to the understanding of the effect of air pollution on mental health in China and confirmed that neighbourhood social capital were protective factors in the relationship between health and environment hazards.

## 1. Introduction

Depressive symptoms are a fundamental problem globally and are considered one of the most severe mental health problems [1]. As of 2010, depressive disorder was the 11th leading cause of disability-adjusted life years globally [2]. Depression is not only associated with physical illnesses like cardiovascular problems [3,4], decreased quality of life [5], and decreased work productivity [6,7], but also increases the mortality and suicide rate [3,4]. Data from the China Health and Retirement Longitudinal Study showed that the rate of depression among the elderly in China reached 31.2% in 2013 [8]. Therefore, depression has become a notable problem in China. Most previous research has indicated that depressive symptoms are significantly related to socioeconomic status and health-related behaviour [2,3,4,5]. For example, people with higher educational attainment and household income are less likely to suffer from depression than those who are less affluent and educated [2,3,4,5]. Similarly, people who drink alcohol or smoke frequently and engage in fewer physical activities are more likely to suffer from depression than those who refrain from drinking, smoking and regularly exercise [2,3,4,5]. 

Recent studies in developed countries have reported that higher air pollution may decrease residents’ mental health, especially increasing the risk of experiencing depression [9,10,11,12,13,14,15,16]. Although the biological mechanisms for this association are not completely understood [12], possible pathological pathways are that: (1) air pollution may increase the risk of cardiovascular illness and thus increases the risk of depression [11,12,17]; and (2) air pollution influences mental health by affecting the nervous and digestive system [12,18]. In addition, another possible biological pathway is that air pollution may reduce the volume of sunlight which may be a stressor for residents’ nervous system and contributes to the development of depression [19,20,21]. Besides biological effects, air pollution may also affect depression through other health-related behaviours. One possible pathway is that air pollution may pose a barrier to involvement with outdoor physical exercises, which is an effective way to cope with the risk of mental health problems [16,22,23,24]. Another explanation is associated with access to face-to-face social contact. Some studies have shown that air pollution discouraged face-to-face social contact among neighbours and therefore increased the risk for depression [25,26,27]. Although a growing body of literature has investigated the health effect of Particulate Matter 2.5 (PM_2.5_) in Chinese cities, this body of research is largely related to the effects on physical health rather than those on mental health [9,10].

Social capital has been defined as a type of resource that provides people with convenience, and it has become essential for the maintenance of population health over the last two decades [28]. Thus, many studies have reported that social capital including social trust, social reciprocity, and social group membership can improve residents’ health, as people living in neighbourhood with higher neighbourhood social capital could more easily acquire emotional or material support from others [1,28]. In recent years, social capital research has posited that neighbourhood social capital may also be protective for mental health, as it weakens the negative influences of neighbourhood social hazards [29,30,31,32,33,34,35,36,37]. For example, Feng et al. have noted that neighbourhood social trust can improve residents’ mental health, since people in communities with high neighbourhood social trust can get more health information [31]. Lindström et al. have pointed out that neighbourhood social group membership and participation may benefit residents’ mental health, as residents are more likely to get support from other neighbourhood group members [32]. Abbott et al. have found that neighbourhood social reciprocity may increase people’s health by strengthening social ties and regulating residents’ health related behaviours [33]. Further, neighbourhood social capital may be a buffer between social environmental hazards and residents’ mental health [29,34,35,36,37]. For example, Niedzwiedz et al. have indicated that neighbourhood group membership may act as a buffer between the inequity of household income and older people’s mental health [29]. Murayama et al. have also reported that the presence of both neighbourhood social trust and social reciprocity can weaken the negative effects of neighbourhood social environment on residents’ depressive mood [37]. However, other researchers have recently argued that social capital may not only act as a protective factor for social environmental hazards but also for physical environmental hazards [38]. For instance, social capital may moderate the effect of industrial pollution exposure on residents’ self-reported health because air pollution may contribute to feelings of stress in residents, and social capital can be a buffer its effects [38].

**Figure 1:**
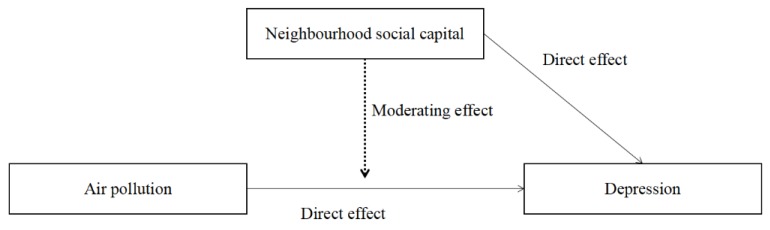
Conceptual model of the moderating relationship of neighbourhood social capital on the relationship between air pollution and depressive symptoms.

Although earnest efforts have been adopted to investigate the relationship between air pollution and depressive symptoms in developed countries, the relationship is still unclear in developing countries, such as China. Furthermore, although there is a consensus that neighbourhood social capital is beneficial for mental health, its protective benefits in the relationship between depressive symptoms and air pollution is still unclear. To bridge these gaps, this study investigated the relationship between PM_2.5_ concentrations and depressive symptoms in China using data from the 2016 wave of the China Labourforce Dynamics Survey (CLDS 2016). We further examine whether neighbourhood social capital has a protective influence on the relationship between depressive symptoms and PM2.5 concentrations. This study contributes to the body of literature in two respects: first, it improves our understanding of how air pollution negatively influences people’s mental health in China; and, second, it provides a deeper understanding of the protective function of neighbourhood social capital for mental health. The conceptual model is presented in Figure 1.

Based on the conceptual model and the review of existing literature, we propose the following hypotheses:

**Hypothesis** **1.** *Residents who live in cities with higher levels of air pollution are more likely to have higher levels of depressive symptoms compared to residents in other cities.*

**Hypothesis** **2.** *Residents who live in neighbourhood with higher neighbourhood social capital are likely to have lower levels of depressive symptoms than residents who live in neighbourhood with lower social capital.*

**Hypothesis** **3.** *Neighbourhood social capital weakens the negative effect of air pollution on residents’ depressive symptoms.*

## 2. Data and Methods

### 2.1. Data

The CLDS 2016 conducted by the Centre for Social Science Survey of Sun Yat-sen University [39] was the primary source of the data. Respondents from this survey were chosen by using a probability proportional to size sampling technique. First, 158 prefecture-level divisions from 29 provinces were randomly selected. Second, 401 neighbourhoods were randomly chosen from the prefecture-level divisions. In China, neighbourhoods refer to basic administrative divisions nested within prefectures (prefecture-level division means the second-level administrative divisions). Overall, 20,861 individuals nested within 401 neighbourhoods nested within 158 prefectures were included in the final dataset.

**Figure 2:**
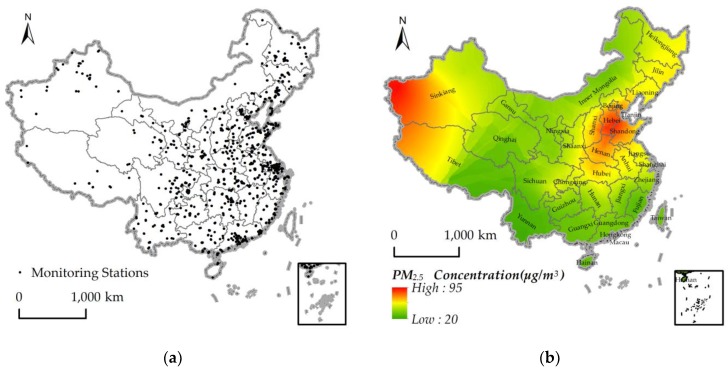
(**a**) Locations of monitoring stations; (**b**) PM2.5 measurement in January 2015.

Average annual PM_2.5_ concentrations of each prefecture were acquired from a real-time remote inquiry website—Airborne Fine Particulate Matter and Air Quality Index [40]—which provides a quantitative hourly index of air pollutants such as AQI, CO, NO_2_, SO_2_, O_3_, PM_10_, and PM_2.5_. By calling the API of the website, the AQI observation data from January to December 2015 in 1613 monitoring stations were collected and cleaned. However, there was a lot of missing data and noise in the observation data. Therefore, a Kalman filter was used for the optimal estimate of PM_2.5_ observation values [41]. A Kalman filter is a time-domain filtering algorithm, which can obtain the optimal estimation of the next moment based on the state of the system and the observation value at the next moment [42]. Another problem was that the recorded data had neither longitude nor latitude information, which may make it difficult to represent spatial variation. To map the data into a geographical space, geocoding was used to associate the observation data with spatial coordinates. Figure 2a shows the locations of 1613 monitoring stations in China in 373 cities. Further, the ordinary kriging interpolation method was used to generate a continuous raster surface (pixel size = 500 m), and zonal statistics was applied to extract the mean concentrations of PM2.5 in each prefecture. Figure 2b shows the spatial interpolation of the average PM_2.5_ concentration in 2015, which was obtained by applying the Kriging method to the ground-based observations average in 2015.

### 2.2. Variables

The Center for Epidemiologic Studies Depression scale (CES-D) was used to calculate depressive symptoms, which has been widely used in previous studies [1]. The CES-D has a Cronbach’s alpha value of 0.95. Independent variables included PM_2.5_ concentrations and social capital indicators. Following existing studies ,the average annual PM_2.5_ concentrations of each prefecture were used to measure the severity of air pollution in cities in 2015 [11,12].

Based on the indicators used in previous studies, three main social capital indicators (i.e social trust, social reciprocity, and social group membership) were included [28]. These three social capital indicators were measured based on variables included in the questionnaire (Part six: Social participation and support) that was collected in CLDS 2016. The question ‘Would you say most people can be trusted?’ was used to measure respondents’ perceptions of social trust. The response ‘*Neighbours are extremely/very trustworthy*’ was defined as high social trust. The question ‘Would you say most of the time people try to be helpful?’ was used to assess respondents’ perceptions of social reciprocity. The response ‘*Neighbours always/often help each other*’ was defined as high social reciprocity. Lastly, respondents were asked about membership in a various kind of voluntary groups. Following existing studies, the percentage of respondents being high trust and high reciprocity within each neighbourhood were calculated and defined as aggregated neighbourhood social trust and aggregated neighbourhood social reciprocity [22]. Furthermore, the average number of types of voluntary groups within each neighbourhood was calculated and defined as aggregated neighbourhood social group membership.

Lastly, we controlled for a series of individual-level and neighbourhood-level variables including: gender (dichotomous variable), age (continuous variable), marital status (categorical variables), educational attainment (categorical variables), employment status (dichotomous variable), *hukou* status (dichotomous variable), living area (dichotomous variable), smoking history (dichotomous variable), drinking history (dichotomous variable), medical insurance status (dichotomous variable), physical status (dichotomous variable), weekly physical exercise time (continuous variable), annual household incomes per capita (continuous variable), and annual neighbourhood incomes per capita (continuous variable). Table 1 shows the summary statistics of variables in the regression models. 

### 2.3. Statistical Analyses

We examined the effects of cities’ PM_2.5_ concentrations on respondents’ depressive symptoms in China while we also tested whether neighbourhood social capital moderated the effect of PM_2.5_ concentrations on respondents’ depressive symptom, using three-level linear regression analyses. Due to the hierarchical structure of this data set, multilevel models were suitable for this research.

We applied hierarchical liner regression analyses to estimate the effect of PM_2.5_ concentrations on depressive symptoms and the moderating effect of neighbourhood social capital. Models presented here are as follows: a baseline model estimating the effect of controlled variables on depressive symptoms (Model 1), a model estimating the effect of neighbourhood social capital on depressive symptoms (Model 2), a model estimating the effect of PM_2.5_ concentrations on depressive symptoms (Model 3), and a model adding cross-level interaction variables into Model 3 while following the multilevel analysis research (Model 4) [43]. All continuous variables were centred on the grand mean in interaction part. The statistical models were of the following form and the mean value of the variance inflation factor (VIF) was less than 3:

(1) $$\begin{array}{ll} CES-D_{ihj}=\beta_{0} & +\beta_{1}Neighbourhood\;social\;capital\;indicators_{hj}+\beta_{2}PM_{2.5}\;concentrations_{j}\\ & +\beta_{3}Neighbourhood\;social\;capital\;indicators_{hj}\cdot PM_{2.5}\;concentrations_{j}\\ & +\beta_{4}Covariates_{h}+\beta_{5}Covariates_{ihj}+\varepsilon_{ihj}+\mu_{hj}+\varphi_{j} \end{array}$$

where i represents individuals, h represents neighbourhoods, and j represents prefectures. $\beta_{0}$ is the intercept. $Neighbourhood\;social\;capital\;indicators_{hj}$ represents a vector of neighbourhood-level variables of social capital. $PM_{2.5}\;concentrations_{j}$ represents a vector of prefecture-level variables of $PM_{2.5\;}concentration$. ${\mathrm{Social}\;\mathrm{capital}\;\mathrm{indicators}}_{\mathrm{hj}}\;\cdot PM_{2.5}{\;\mathrm{concentrations}}_{j}$ represents a vector of cross-level interaction effect. $Covariates_{hj}$ represent a vector of neighbourhood-level covariates. $Covariates_{ihj}$ represent a vector of individual-level covariates. $\varepsilon_{ihj}$, $\mu_{hj}$, $\varphi_{j}$ represent random errors at the individual level, neighbourhood level, and city level, respectively. The interaction effect of interest is expressed in coefficients $\beta_{3}$.

## 3. Results

Table 2 shows the results of the multilevel linear models on respondents’ depressive symptoms. Model 1 included individual-level and neighbourhood-level control variables. Compared with women, men had a lower CES-D score (coefficient = −1.243, standard error = 0.153). In addition, respondents’ CES-D scores increased with age (coefficient = 0.040, standard error = 0.005). Married respondents had a lower CES-D score (married and living with a spouse, coefficient = −1.052, standard error = 0.179; married and living apart with a spouse, coefficient = −0.718, standard error = 0.264). Respondents with higher educational attainment had a lower CES-D score, compared with respondents who graduated from primary school or below, (high school, coefficient = −1.065, standard error = 0.153; college and above, coefficient = −1.064, standard error = 0.251). Compared with unemployed respondents, employed respondents have lower CES-D scores (coefficient = −0.558, standard error = 0.265). What’s more, respondents with medical insurance had lower CES-D scores (coefficient = −0.850, standard error = 0.203) than those without medical insurance. Respondents with physical diseases have higher CES-D scores (coefficient = 5.897, standard error = 0.196) than those without physical diseases. Furthermore, respondents’ CES-D scores decreased with physical exercise time (coefficient = −0.119, standard error = 0.026). Interestingly, respondents’ CES-D scores decreased with the logarithm of household incomes and neighbourhood incomes (logarithm of household incomes, coefficient = −0.579, standard error = 0.064; logarithm of neighbourhood incomes, coefficient = −1.150, standard error = 0.311). Lastly, CES-D scores decreased with all three individual-level social capital indicators (neighbours are extremely/very trustworthy, coefficient = −1.736, standard error = 0.148; neighbours always/often help each other, coefficient = −1.133, standard error = 0.131; number of types of voluntary groups, coefficient = −0.261, standard error = 0.131). Model 2 included control variables and neighbourhood social capital indicators.

Surprisingly, respondents’ CES-D scores decreased with all of neighbourhood social capital indicators (neighbourhood social trust, coefficient = −4.152, standard error = 1.443; neighbourhood social reciprocity, coefficient = −1.959, standard error = 0.841; neighbourhood social group membership, coefficient = −0.968, standard error = 0.489). Model 3 included control variables, social capital indicators, and PM_2.5_ concentrations. The results showed that respondents’ CES-D scores increased with logarithm of PM_2.5_ concentrations (coefficient = 2.167, standard error = 1.090) which means PM_2.5_ concentrations had significant negative effect on respondents’ mental health. Lastly, cross-level interaction effects were added in Model 4. The effect of PM_2.5_ concentrations on respondents’ CES-D scores varied by neighbourhood social capital indicators which means that neighbourhood social capital significantly moderated the relationship between city’s PM_2.5_ concentrations and respondents’ depressive symptoms.

**Figure 3:**
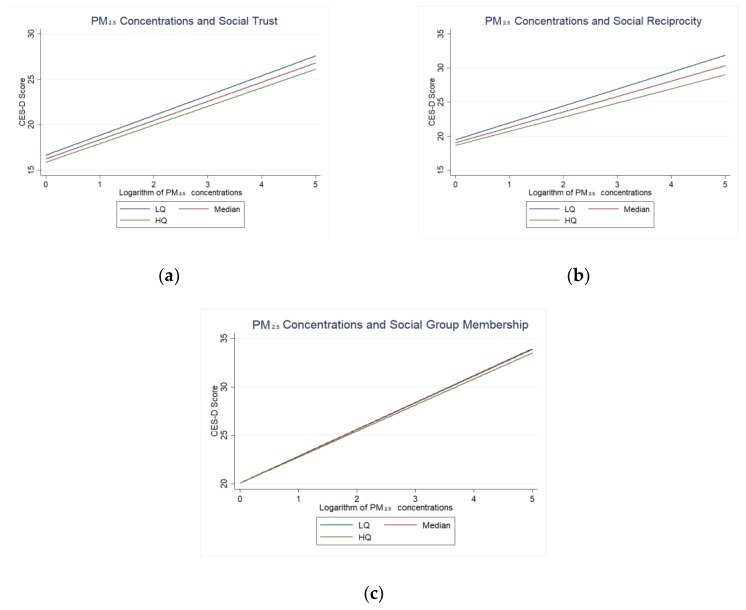
(**a**) Predicted relationship between PM_2.5_ concentrations and CES-D scores differing by social trust; (**b**) Predicted relationship between PM_2.5_ concentrations and CES-D scores differing by social reciprocity; (**c**) Predicted relationship between PM_2.5_ concentrations and CES-D scores differing by social group membership.Lower Quartile (LQ = 25%), the median (MQ = 50%) and higher quartile (HQ = 75%).

Figure 3 graphically displays the predicted PM_2.5_ concentrations-depressive symptoms differing by neighbourhood social capital indicators in Model 4, where different neighbourhood social capital indicators are represented by the Lower Quartile (LQ = 25%), the median (MQ = 50%) and higher quartile (HQ = 75%). The result of Model 4 and Figure 3a shows that respondents living in cities with higher concentrations of PM_2.5_ with higher neighbourhood social trust had lower CES-D scores than respondents living in cities with higher concentrations of PM_2.5_ with lower neighbourhood social trust. With the rise of neighbourhood social trust, its moderating effect was strengthened. Figure 3b shows that respondents living in cities with higher concentrations of PM_2.5_ with higher neighbourhood social reciprocity had lower CES-D scores than did respondents living in cities with higher concentrations of PM_2.5_ with lower neighbourhood social reciprocity. With the rise of neighbourhood social reciprocity, its moderating effect was strengthened. 

Figure 3c shows that respondents living in cities with higher concentrations of PM_2.5_ with higher neighbourhood social group membership had lower CES-D scores than respondents living in cities with higher concentrations of PM_2.5_ with lower neighbourhood social group membership. With the rise of neighbourhood social group membership, its moderating effect was strengthened; however, the moderating effect of the difference of median (MQ = 50%) and higher quartile (HQ = 75%) of neighbourhood social group membership is not obvious.

## 4. Discussion

In the present study, we investigated the relationship between PM_2.5_ concentrations and depressive symptoms in China. First, as has been demonstrated in previous studies respondents’ depressive symptoms increased with cities’ PM_2.5_ concentrations [11,13,14,16]. Such a finding may be due to the following reasons: (1) air pollution may increase the risk of cardiovascular diseases, and cardiovascular diseases are closely related to depressive symptoms [17,44]; therefore, residents living in cities with more air pollution are more likely experience both cardiovascular diseases and depressive symptoms, thus continuing a vicious circle. (2) Air pollution may also decrease the frequency of residents’ outdoor physical activities, and outdoor physical activities are associated with depressive symptoms [38,39,45,46].

In addition, previous studies we found that neighbourhood social capital was beneficial to residents’ health [1,28,30,47]. Social capital can increase access to local services and amenities [28] and provide useful support for residents [28]. Rapid urbanization in China has eroded residents’ connection with friends and relatives [48]. Therefore, neighbourhood social capital has become essential to residents’ health in China. For this reason, neighbourhood social capital benefits residents’ mental health in China.

Most importantly, the statistical significance of the interaction effect indicates that neighbourhood social capital can weaken the negative impact of PM_2.5_ concentrations on depressive symptoms. In other words, neighbourhood social capital exerts a protective effect on the relationship between depressive symptoms and PM_2.5_ concentrations. There are several explanations for the protective effect. First, as for neighbourhood social trust, previous studies have noted that health knowledge spreads faster in high-social-trust neighbourhoods (vs. low) since people are more likely to share health knowledge with others and accept others’ advice [28,30,49,50]. Therefore, residents experiencing depressive symptoms caused by PM_2.5_ may acquire useful health knowledge about cardiovascular, nervous, and digestive system disease prevention more easily and can learn how to cope with stressor from lack of sunlight in a high-social-trust neighbourhood. Second, social interactions are more frequent in high-social-trust neighbourhoods, since their residents are more likely to be in contact with those whom they trust [28,30,49,50]. Thus, although PM_2.5_ concentrations may lead to a decrease in face-to-face social contact among neighbours’ , residents living in high-social-trust neighbourhoods maintain connections with their neighbours through indoor activities (for example, playing card games or Mah-jong). As a result, the negative effect of PM_2.5_ may be weakened by neighbourhood social trust.

In addition, as for neighbourhood social reciprocity, residents can not only obtain emotional support but also can receive material support from neighbours in a high-social-reciprocity neighbourhood [28,30,51,52,53]. Therefore, residents experiencing depressive symptoms caused by PM_2.5_ may garner emotional comfort by talking to their neighbours about their mood or also acquire useful health knowledge of cardiovascular, nervous and digestive system disease prevention and know how to cope with stressor from lack of sunlight. All this will make residents feel less depressed even while still experiencing PM_2.5_.

Furthermore, as for neighbourhood social group membership, living in a high-social-group-membership neighbourhood, residents are more willing to participate in group activities [28,29]. Although PM_2.5_ may decrease residents’ willingness to have physical activities outdoor, living in high-social-group-membership neighbourhood may increase their willingness to have both outdoor and indoor physical activities. Social interactions among group members are more frequent than that among non-group members [28,29]. Even with fewer daily interactions, group members can maintain their interactions by attending routine group activities. In a word, the negative effect of PM_2.5_ is weakened by neighbourhood social group membership.

Lastly, this study also revealed that the protective effect of neighbourhood group membership is relatively weaker than the other two neighbourhood social capital effects, and this may be because this research used the average number of types of voluntary groups within each neighbourhood to measure neighbourhood group membership. However, we failed to measure the strength of the social ties within each group, which means one may be a part of several social groups but fail to garner strong ties in each respective group [28].

From a policy perspective, to decrease depressive symptoms, the government should pay attention to the following three aspects. First, more trees and grass should be planted in cities to increase green space. Green space can benefit residents’ health and reduce air pollution [54,55]. In addition, more public medical insurance and subsidies should be provided to low-income residents to weaken the negative effect of environmental hazards on poor residents’ health. Finally, neighbourhood social capital should be promoted. Existing studies have found that neighbourhood social capital can be improved by promoting residents’ educational attainment; therefore, more funding should be provided to improve national education [56,57,58].

Despite this study’s advantages, some limitations should be noted. First, due to the cross-sectional nature of the data, we were unable to capture time-fixed effects and only calculated average PM_2.5_ concentrations in 2015. Second, we used only PM_2.5_ concentrations as an indicator of air pollution; previous studies have used other air pollution indicators related to ozone, nitrogen dioxide, and sulphur dioxide [11,12]. Lastly, we measured the concentration of PM_2.5_ at the city level; future research should utilize air quality index at a finer geographical level.

## 5. Conclusions

This study confirms that neighbourhood social capital plays a protective role in the relationship between depressive symptoms and PM_2.5_ concentrations in China. PM_2.5_ concentrations may increase respondents’ depressive symptoms, while neighbourhood social capital may decrease respondents’ depressive symptoms. Most importantly, neighbourhood social capital weakens the negative influences of PM_2.5_ concentrations on respondents’ depressive symptoms. However, the mechanism through which air pollution negatively impacts residents’ mental health is still unclear in developing countries. Therefore, further studies are warranted.

## Figures and Tables

**Table 1:** Summary statistics of variables included in regression analyses.

Variables	Proportion/Mean (SD)
**Dependent variables**	
CES-D Score (0–60)	7.3 (9.24)
**Independent variables**	
Neighbourhood social capital	
Neighbourhood social trust	0.78 (0.12)
Neighbourhood social reciprocity	0.48 (0.23)
Neighbourhood social group membership	0.08 (0.15)
PM_2.5_ concentrations ($\mu g/m^{3}$)	49.27 (19.74)
**Control variables**	
Gender	
Male	0.48
Female	0.52
Age	44.83 (14.61)
Marital status	
Single, divorced, and widowed	0.19
Married and living with spouse	0.73
Married but living apart from spouse	0.08
Education	
Primary school or below	0.35
High school	0.52
College and above	0.13
Employment	
Employed	0.95
Unemployed	0.05
Hukou status	
Local *hukou*	0.91
Non-local *hukou*	0.09
Living area	
Living in urban neighbourhood	0.39
Living in rural neighbourhood	0.61
Smoking	
Current smoker	0.27
Non-smoker	0.73
Drinking	
Drinker	0.19
Non-drinker	0.81
Medical insurance	
Having medical insurance	0.90
No medical insurance	0.10
Physical health status	
Have a disease	0.11
No disease	0.89
Weekly physical exercise time (minutes)	97.51 (267.95)
Average annual household incomes per household member (Chinese yuan)	17991.68 (202477.08)
Average annual neighbourhood incomes per neighbourhood resident (Chinese yuan)	17814.06 (3.22)
Individual-level social capital	
Trust in neighbours	
Neighbours are extremely/very trustworthy	0.78
Neighbours are somewhat/slightly/not at all trustworthy	0.22
Neighbours are helpful	
Neighbours always/often help each other	0.48
Neighbours sometimes/seldom/never help each other	0.52
Number of types of voluntary groups	0.08 (0.37)

**Table 2:** Multilevel liner regression coefficients for the effects of PM2.5 concentrations, social capital, and individual characteristics on depressive symptoms.

Effects and Variables	Model 1 (Baseline)	Model 2	Model 3	Model 4
Fixed part				
Logarithm of PM_2.5_ concentrations			2.167 ** (1.090)	2.670 ** (1.390)
Neighbourhood-level social capital				
Neighbourhood social trust		−4.152 *** (1.443)	−4.247 *** (1.443)	−4.271 *** (1.498)
Neighbourhood social reciprocity		−1.959 *** (0.841)	−1.878 *** (0.854)	−2.083 *** (0.849)
Neighbourhood social group membership		−0.968 ** (0.489)	−0.962 ** (0.481)	−0.146 ** (0.073)
Male (ref: female)	−1.243 *** (0.153)	−1.240 *** (0.153)	−1.240 *** (0.153)	−1.240 *** (0.153)
Age	0.040 *** (0.005)	0.040 *** (0.005)	0.040 *** (0.005)	0.040 *** (0.005)
Marital status and family organization(ref: single, divorced, and widowed)				
Married and living with spouse	−1.053 *** (0.179)	−1.050 *** (0.179)	−1.050 *** (0.179)	−1.050 *** (0.179)
Married but living apart from spouse	−0.718 *** (0.264)	−0.719 *** (0.264)	−0.718 *** (0.264)	−0.719 *** (0.264)
Education (ref: primary school or below)				
High school	−1.065 *** (0.153)	−1.075 *** (0.153)	−1.077 *** (0.153)	−1.074 *** (0.153)
College and above	−1.064 *** (0.251)	−1.078 *** (0.252)	−1.081 *** (0.252)	−1.082 *** (0.252)
Employed (ref: unemployed)	−0.558 ** (0.265)	−0.552** (0.265)	−0.553 ** (0.265)	−0.553 ** (0.265)
Local hukou (ref: non-local hukou)	−0.324 (0.246)	−0.273 (0.247)	−0.273 (0.247)	−0.279 (0.247)
Living in urban neighbourhood(ref: living in rural neighbourhood)	0.108 (0.345)	−0.240 (0.424)	−0.243 (0.424)	−0.223 (0.427)
Current smoking status (ref: non-smoker)	0.100 (0.173)	0.091 (0.173)	0.092 (0.173)	0.088 (0.173)
Current drinking status (ref: non-drinker)	−0.057 (0.171)	−0.056 (0.171)	−0.056 (0.171)	−0.055 (0.171)
Medical insurance (ref: no medical insurance)	−0.850 *** (0.203)	−0.848 *** (0.203)	−0.848 *** (0.203)	−0.846 *** (0.203)
Have a disease (ref: no disease)	5.897 *** (0.196)	5.886 *** (0.196)	5.887 *** (0.196)	5.889 *** (0.196)
Logarithm of physical exercise time	−0.119 *** (0.026)	−0.120 *** (0.026)	−0.120 *** (0.026)	−0.120 *** (0.026)
Logarithm of household incomes per capita	−0.579 *** (0.064)	−0.588 *** (0.064)	−0.588 *** (0.064)	−0.587 *** (0.064)
Logarithm of neighbourhood incomes per capita	−1.150 *** (0.311)	−1.279 *** (0.315)	−1.287 *** (0.316)	−1.206 *** (0.316)
Individual-level social capital				
Neighbours are extremely/very trustworthy (ref: neighbours are somewhat/slightly/not at all trustworthy)	−1.736 *** (0.148)	−1.698 *** (0.149)	−1.698 *** (0.149)	−1.698 *** (0.149)
Neighbours always/often help each other (ref: neighbours sometimes/seldom/never help each other)	−1.133 *** (0.131)	−1.094 *** (0.133)	−1.094 *** (0.133)	−1.095 *** (0.133)
Number of types of voluntary groups	−0.261 ** (0.131)	−0.250 ** (0.125)	−0.250 ** (0.125)	−0.250 ** (0.125)
Cross-level interaction				
Neighbourhood social trust × logarithm of PM2.5 concentrations				−0.846 ** (0.401)
Neighbourhood social reciprocity × logarithm of PM2.5 concentrations				−1.019 *** (0.102)
Neighbourhood social group membership × logarithm of PM2.5 concentrations				−0.924 ** (0.437)
Constant	16.929 *** (0.756)	20.021 *** (1.335)	19.482 *** (2.018)	19.111 *** (1.998)
Random part				
Var (city-level constant)	2.020 ***	2.210 ***	2.190 ***	1.990 ***
Var (neighbourhood-level constant)	5.192 ***	4.922 ***	4.916 ***	4.917 ***
Var (Residual)	70.301 ***	71.299 ***	70.300 ***	70.300 ***
Number of cities	158	158	158	158
Number of neighbourhoods	401	401	401	401
Number of individuals	20,861	20,861	20861	20,861
AIC	148,666.800	148,664.500	148,662.300	148,660.500

** *p* < 0.05, *** *p* < 0.01. All continuous independent variables and covariates were grand-mean centred.
